# Pulmonary Complications in Patients with Severe Brain Injury

**DOI:** 10.1155/2012/207247

**Published:** 2012-10-23

**Authors:** Kiwon Lee, Fred Rincon

**Affiliations:** ^1^Mischer Neuroscience Institute, Memorial Hermann-Texas Medical Center, 6431 Fannin Street Medical School Building 7152, Houston, TX 77030, USA; ^2^The Vivian L. Smith Department of Neurosurgery, 6431 Fannin Street, Room 7.152, Houston, TX 77030, USA; ^3^Divisions of Critical Care and Neurotrauma, Departments of Neurology and Neurosurgery, Thomas Jefferson University, Philadelphia, PA 19107, USA

## Abstract

Pulmonary complications are prevalent in the critically ill neurological population. Respiratory failure, pneumonia, acute lung injury and the acute respiratory distress syndrome (ALI/ARDS), pulmonary edema, pulmonary contusions and pneumo/hemothorax, and pulmonary embolism are frequently encountered in the setting of severe brain injury. Direct brain injury, depressed level of consciousness and inability to protect the airway, disruption of natural defense barriers, decreased mobility, and secondary neurological insults inherent to severe brain injury are the main cause of pulmonary complications in critically ill neurological patients. Prevention strategies and current and future therapies need to be implemented to avoid and treat the development of these life-threatening medical complications.

## 1. Introduction

Pulmonary complications are very prevalent in the critically-ill neurological population. Respiratory failure, pneumonia, pleural effusions and empyema, acute lung injury and the acute respiratory distress syndrome (ALI/ARDS), pulmonary edema, and pulmonary embolism (PE) from venous thromboembolism (VTE) are frequently encountered in this patient population [1–7]. In addition, direct chest trauma and patients with traumatic brain injury (TBI) are not exempt from direct complications such as rib fractures, flail chest, lung contusions, and hemo/pneumothorax. Unfortunately, the development of these complications extends the patient's need for care in the intensive care unit (ICU) and prevents early mobilization, and this increases the likelihood of developing secondary disability.

Direct brain injury, depressed level of consciousness and inability to protect the airway, disruption of natural defense barriers, decreased mobility, and secondary physiopathologic insults inherent to severe brain injury are the main cause of pulmonary complications in critically-ill neurological patients. The goal in the ICU is to prevent, treat, and optimize hypoxemia and maintain oxygen delivery to limit secondary neurological insults. In the absence of feasible pharmacological agents to target these goals, prevention strategies to minimize pulmonary complications such as use of bedside techniques such as thoracentesis, closed thoracostomies (chest tubes), lung-protective ventilator strategies, bundles for prevention of ventilator associated pneumonias (VAP), and deep venous thrombosis (DVT) prophylaxis are the cornerstone in the prevention and management of pulmonary complications in severe brain injured patients. 

Finally, additional strategies to target physiopathological end-points such as inflammation may need to be developed, studied in clinical trials, and deployed to clinical practice, to optimize the outcomes in this patient population. This paper summarizes the most important pulmonary complications encountered in the critically-ill neurological population.

## 2. Pulmonary Complications Related to Direct Chest Trauma

**Figure 1:**
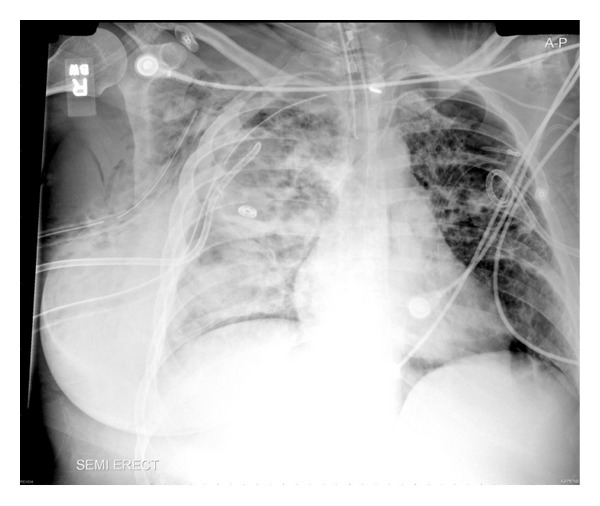
Chest X-ray of an ARDS victim who has developed multiple pneumothoraces secondary to a bronchopleural fistula.

Patients who sustain TBI are often at risk for the development of other traumatic injuries such as rib fractures, lung contusions, flail chest, and pneumo/hemothorax. The implementation of a routine standardized assessment of the traumatized victim provides a highly sensitive protocol to diagnose these injuries [8]. A traumatic pneumothorax, defined as the entry of air into the pleural space, occurs after both penetrating and nonpenetrating thoracic injuries. A simple pneumothorax occurs when there is no communication with the external environment or any shift of mediastinal structures (Figure 1), an open pneumothorax occurs when a communication or fistula exists between the pleural space and the environment (sucking wound), and finally, a tension pneumothorax occurs when escape of pleural air to the environment is prevented, and increasing intrapleural pressure leads to shift in mediastinal structures with associated hemodynamic compromise. Treatment of a small pneumothorax in a traumatized victim undergoing positive pressure ventilation requires the use of chest tubes, and a conservative approach with normobaric hyperoxia is not an alternative. However, patients with blunt trauma breathing spontaneously and with occult pneumothoraces could be safely observed [9]. Open pneumothoraces require (a) chest tube, (b) mechanical ventilation, and (c) immediate surgical repair of the wound. Treatment of tension pneumothorax requires the use of immediate decompression (needle thoracostomy) and/or rapid placement of a chest tube. The persistence of air leak and pneumothorax is indicative of a bronchopleural fistula and therefore requires immediate surgical revision with thoracotomy (Figure 1).

A hemothorax is the accumulation of blood in the pleural space and may be the cause of respiratory distress, pain, hypoxia, and circulatory arrest. A massive hemothorax is defined as the presence of more than 1000 cc of blood or the chest tube output of more than 200 cc/h [8]. The treatment of a hemothorax requires: (a) restitution of circulatory blood volume if needed, (b) oxygen supply and restoring the airway, and (c) closed thoracostomies (chest tubes).

A flail chest results when three or more adjacent ribs are fractured at two different points, allowing a freely moving segment of the chest wall. This pattern of fractures is often associated with severe pain, underlying pulmonary contusions, and respiratory failure secondary to paradoxical movements of the chest wall. Most of the times patients require mechanical ventilation and pain control but the decision to intubate may require individualization.

## 3. Respiratory Failure and Pneumonia

 Neurologic related respiratory failure from severe central nervous system dysfunction is one of the most frequent reasons for initiating mechanical ventilation [10]. Among the causes of neurologic dysfunction, structural causes such as ischemic stroke (AIS), hemorrhages (intracerebral hemorrhage (ICH) and subarachnoid hemorrhage (SAH)), and traumatic brain injury (TBI) carry the worst prognosis and are the greatest challenge to critical care specialists based on the interaction between hypoxemia and secondary neurological insults.

In a recently published retrospective multicenter cohort study from a prospective compiled and maintained registry, Pelosi et al. studied the epidemiology, clinical characteristics, and clinical practices in relation to mechanical ventilation in a cohort of critically-ill neurological patients. Though SAH patients were excluded, this study is an excellent description of day-to-day practices across different types of ICUs around the globe. Not surprisingly, neurological patients had lower Glasgow Coma Scale (GCS) on admission, more ICU and ventilator-days, had more early tracheostomies, more VAP rates, but more interestingly, the rate of reintubation was similar to those of nonneurological patients. In this sense, this study provides support that mental status and GCS may not matter at the time of extubation [11], as GCS was higher in nonneurological patients and the rate of reintubation was the same. Though the Pelosi study is important [1], it does not answer the question of which neurological patients are more likely to get “stuck” on the ventilator or need early reintubation. The interaction with disease severity, age, neurological diagnosis, and important variables that worry all critical care specialists at the time of extubation such as characteristics and management of secretions, development of atelectasis due to hypoventilation [12], cranial nerve involvement (pupillary abnormalities, absence of gag, etc.) are missing in this analysis. These questions may need to be answered in different prospective clinical trials, but in the meantime, clinical expertise may need to guide the best approach to a particular patient.

Pneumonia is a common complication of severe brain injury and can occur in up to 60% of patients [13] as these patients are prone to aspirate stomach contents. Similarly, VAP is a preventable secondary consequence of prolonged intubation and mechanical ventilation. VAP is pneumonia that develops in an intubated patient after 48 hours or more of ventilatory support [14]. Critically-ill neurological patients that are mechanically ventilated are at an increased risk of VAP due to factors such as decreased level of consciousness; dry, open mouth; microaspiration of secretions [15]. Patients with severe brain injury tend to be on mechanical ventilation longer than medically intubated patients, and VAP in the neurologic ICU can further increase the length of stay (LOS) [16]. In patients with severe ischemic stroke, the development of VAP is associated with a 3-fold increase of in-hospital mortality [17]. The implementation of VAP bundles including oral care has been shown to decrease the rate of VAP in critically-ill neurological patients [18]. Additional measures to decrease VAP include daily vacation sedation to examine readiness for extubation, management of upper airway secretions with closed aspiration systems, and strict control of endotracheal-tube cuff pressures, policies related to hand hygiene, head elevation at 45°, oral hygiene with chlorhexidine preparations, along with stress ulcer prophylaxis with H2 or proton-pump inhibitors [19].

Though neurological patients experience more early tracheotomies in general [1], this practice has not been associated with improved patient outcomes, particularly mortality or onset of VAP [20, 21]. Accumulation of fluid in the pleural space and bacterial infection may result in empyema. Treatment of empyema and complicated pleural effusions requires evacuation of the infected material via chest tube and antibiotic regimen.

## 4. ALI and ARDS

**Figure 2:**
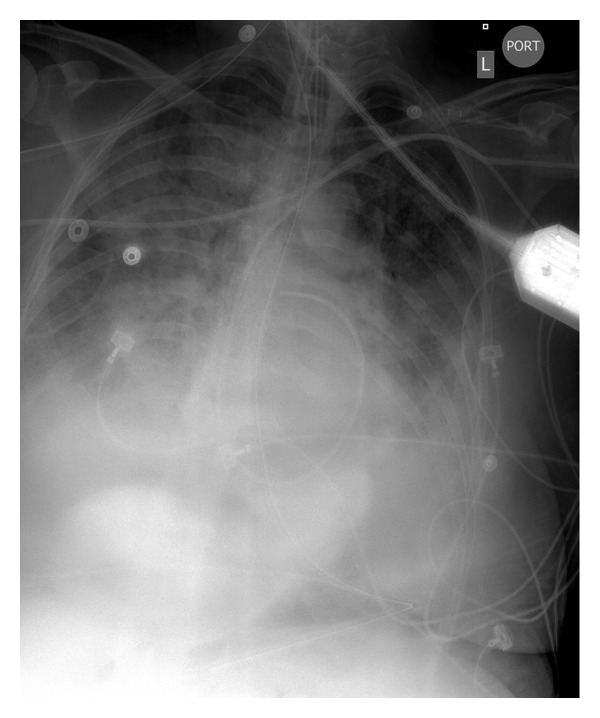
Chest X-ray of an ARDS victim that suffered a grade 4 SAH.

ALI and the more severe form of lung injury, the ARDS, are a continuum of inflammatory responses following direct or indirect insults to the lung and clinically recognized by the onset of hypoxemia, reduced pulmonary compliance, and radiographic appearance of bilateral infiltrates [22]. The incidence of ALI/ARDS syndrome has been reported in 20–25% of patients with isolated traumatic brain injury (TBI) [23, 24]. In patients with SAH an incidence of 20–30% has been reported as well [2, 3], and in acute ischemic stroke (AIS), a recent epidemiological study reported that the cumulative incidence of ARDS from 1994 to 2008 was 4% [6]. In all reports, the mortality and outcomes are substantially the worst [3, 6, 24] (Figure 2).

**Figure 3:**
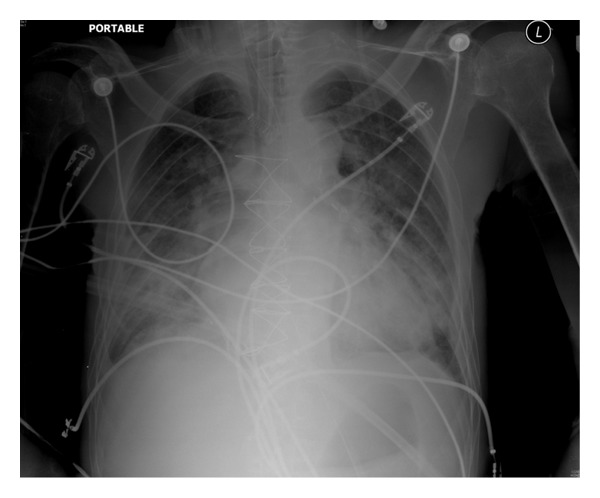
Pulmonary edema from volume overload as complication of Triple H Therapy in a patient with Grade 4 SAH. Note distended pulmonary arteries and prominent cardiac silhouette.

ARDS is defined as a syndrome characterized by acute onset of bilateral lung infiltrates consistent with pulmonary edema (Figure 3), absence of signs of left atrial hypertension (usually a pulmonary artery occlusion pressure (PAOP) of < 18 mmHg), and hypoxemia with a PaO2/FiO2 ratio of < 200. Patients with these criteria but with PaO2/FiO2 ratios < 300 are classified as ALI [22]. Risk factors for the development of ALI/ARDS in brain injured patients are the severity of the initial brain injury (lower GCS scores), in-hospital induced hypertension [25], and extracranial factors such as younger age, male gender, white race ethnicity, history of HTN, DM, and COPD, and the development of sepsis has been implicated as well [6, 24].

The physiopathology of ARDS/ALI is rather complex. Initial studies suggested that the development of NPE in patients with TBI [26] and hemorrhagic stroke wash more frequent in those patients who had higher intracranial pressures (ICP) and low cerebral perfusion pressures (CPP, mean arterial pressure (MAP-ICP) [27]. The importance of these landmark studies is that the development of NPE occurred in the absence of clear lung injury and normal chest X-ray (CXR) on admission, suggesting that brain injury was a risk factor for this phenomenon. Explanations for these observations were based on what is known as the “blast injury,” which explains that a surge in adrenergic response is translated into increased capillary pressures in the lung bed, endothelial damage, and subsequent capillary leak into the alveoli and pulmonary interstitium [28]. In addition, inflammatory responses related to the production of mediators such as IL-6 may explain the development of NPE [29, 30]. To this end, a “double hit” model has been proposed where patients suffering severe brain injury experience a “first hit” with an adrenergic surge and systemic production of inflammatory mediators, making the lung more susceptible to injury, and a “second hit” from extracorporeal variables such as infections, transfusions, and mechanical ventilation [31].

The role of mechanical ventilation in the physiopathology of ALI/ARDS has been studied extensively. The onset of systemic inflammation in the setting of brain injury coupled with conventional modalities of mechanical ventilation used in the management of brain injury such as hyperventilation for permissive hypocapnia may be associated with more lung injury [32]. The use of ventilator modalities to achieve mild permissive hypocapnia (PaCO_2_ 30–35 mmHg) may be associated with the use of tidal volumes (Tv) larger than 6–8 ml/kg, which have been associated with the ventilator-induced ling injury (VILI), a syndrome indistinguishable from ARDS [33] and related to overdistention during mechanical ventilation (volutrauma), recruitment-derecruitment of collapsed alveoli (atelectrauma), and activation of inflammatory processes (biotrauma) [31]. 

Conventional ventilatory support in severe brain injured patients relies on the use of assist-control ventilation [1]. Most practitioners would aim at ventilating with low tidal volumes (6–8 ml/kg of PBW), plateau pressures < 30 cm H_2_O, and PEEP levels of 5–10 cmH_2_O, which may be considered controversial [34]. In certain cases and refractory hypoxemia rescue ventilation with higher positive end expiratory pressure (PEEP), prone positioning and recruitment, airway pressure release ventilation (APRV), high frequency oscillation (HFOV), tracheal gas insufflation (TGI), extracorporeal membrane oxygenation (ECMO), and CO_2_ removal (AV-ECCO_2_R) may need to be instituted [31, 34]. Recent observational studies have demonstrated that practitioners may use lower PEEP levels in neurological patients [35], perhaps of fear of affecting intracranial pressures (ICPS). Application of PEEP in brain injury may be associated with three responses: increase or decrease on ICP or no change at all, depending on the end result at the lung and gas exchange level. If PEEP induces alveolar recruitment, a reduction in PaCO_2_ would be seen, with a decrease in ICP. If PEEP induces alveolar hyperinflation only and no net effect on ventilation, there could be an increase in the PaCO_2_, with a concurrent increase in ICP [36]. Lastly, the application of PEEP in those patients in whom alveolar recruitment occurs, but the predominant effect is an improvement in oxygenation rather than a prominent decrease in PaCO2 due to a reduction in dead space, a no significant change in ICP would be expected [36]. At this time, it seems that the use of PEEP to treat ALI/ARDS may be appropriate in the patient with severe brain injury, provided that MAP is maintained and close attention given to ICP and CPP as changes are made. Several authors advocate for determination of cerebral autoregulation to determine if patients may tolerate abrupt changes in PEEP that could result in increased ICP, in those with lost cerebral autoregulation [37]. Finally, the use of steroids in the proliferative phase of ARDS is of questionable use [35, 38–40].

Experimental animal models of induced hypothermia in conjunction with lower ventilatory frequencies have demonstrated the improvement of many variables of acute lung injury such as neutrophil counts and inflammatory markers, suggesting that the role of hypothermia in the management of ALI/ARDS may be studied in clinical trials, and newer applications in the management of ALI/ARDS may be forthcoming [41].

## 5. Pulmonary Edema in the SAH Population

Symptomatic cerebral vasospasm and delayed cerebral ischemia continue to be a major etiology for significant morbidity for patients suffering from acute, aneurysmal subarachnoid hemorrhage (SAH) [42] (Figure 3). Triple H therapy, which consists of hypertension, hemodilution, and hypervolemic therapy, has been a mainstay of the medical therapy for treating symptomatic vasospasm for the past few decades [43]. Each component of this therapy is geared towards augmenting cerebral blood flow (CBF) and perfusion pressure of the brain. Despite a paucity of evidence for indications, triple H therapy has been widely used around the world as both prophylaxis and treatment for cerebral vasospasm. The timing of triggering the initial therapy as well as how each component is used depends primarily on the need of each patient since SAH can be a heterogeneous (i.e., low versus high grade SAH has a dramatically different natural course of illness) and dynamic disease. However, even for the same patient scenario, there is a wide variety of practice patterns in using this therapy, mainly due to lack of evidence for guiding clinicians. According to a recently published survey, most neurointensivists initiated triple H therapy in order to treat symptomatic vasospasm [44]. Therefore, most of the controversies existed in the setting of prophylactic use. While the data for or against such prophylactic use is limited, literature indicates that there is no difference in outcomes between placebo and triple H therapy when used as a prophylaxis against cerebral vasospasm [45, 46]. Regardless of whether it is used as a prophylaxis or treatment for active vasospasm, hypervolemia and hemodilution frequently lead to medical complications most often as pulmonary edema and anemia, which could be associated with worst outcomes in SAH patients [47]. Intravascular volume expansion and targeting a certain level of hemoglobin may provide augmentation of cardiac output and therefore ultimately improve the delivery of oxygen. However, the optimal target for hemoglobin is still unknown but microdialysis studies have shown than in the setting of vasospasm hemoglobin levels lower than 9 mg/dL may be associated with metabolic crisis in the injured brain [48]. The potentially positive effect of hemodilution, especially while ischemic injury is ongoing, may be provided by triple H therapy, but there is also a ceiling effect. No matter how much preload is expanded, the cardiac output does not infinitely increase and thus reaches a certain level of plateau. Oxygen carrying capacity also has the same limitation. Furthermore, in order for the injured and actively ischemic brain to receive adequate oxygen, gas exchange in the lungs must occur optimally. This rather simple, and yet important physiologic concept is often overlooked as emphasis on saving the brain and allowing lung injury such as pulmonary edema is often done. Treating physicians need to understand that there will be a fine balance between optimizing the CBF with hemodilution and the extent of concurrent pulmonary complications, which may adversely affect the ultimate goal of delivery of oxygen and flow to the injured brain. This concept has led to more emphasis on hypertension [49] while ensuring an euvolemic state rather than creating an overly hypervolemic state by arbitrarily using endpoints or surrogates of intravascular volume status [50]. Even today, many centers have central venous pressure (CVP) targets of greater 10 mmHg and pulmonary capillary wedge pressure greater than 12 mmHg as hemodynamic end points. It turns out neither of these variables is accurate surrogate for assessing intravascular volume status and has led to unacceptably high incidence of pulmonary edema which again compromises the ultimate delivery of oxygen to the ischemic brain (Figure 3). A recent systematic review of analyzing CVP has demonstrated a poor relationship between CVP and intravascular volume status and reports that increasing the value of CVP to an arbitrarily determined value does not lead to a positive hemodynamic response [51]. The debate regarding the use of triple H therapy and fine balance between maximizing CBF in the setting of pulmonary edema may continue, and the answer may be similar to many other controversial topics: individualized, case-by-case decisions. In any event, the principle of adequate gas exchange needs to be conveyed and achieved in order to successfully optimize brain oxygenation. Current guidelines for the management of SAH support the maintenance of euvolemia rather than hypervolemia [52].

## 6. Neurogenic Pulmonary Edema

Neurogenic pulmonary edema (NPE) has been reported with a number of proposed mechanisms. While the exact locations and circuits involved in the central nervous system (CNS) have not been clearly identified, this uncommon but potentially life-threatening condition may occur in the setting of acute, severe brain injuries including traumatic brain injury, intracerebral hemorrhage (ICH), and even in seizures. Sudden rise in intracranial pressure (ICP) such as in SAH or ICH, hypothalamic involvement, rapidly occurring sympathetic surge, increased systemic vascular resistance (SVR) have all been implicated in pathophysiology [53]. Elevated tone of venous circulation results in more venous return. Increase in hydrostatic pressure in the pulmonary vasculature may lead to interstitial edema formation [31]. 

Complicating the picture further, elevation in SVR raises afterload for the heart, which in turn can lead to a similar pathophysiology as cardiogenic pulmonary edema with worsening left ventricular failure and further edema formation in the pulmonary interstitial spaces. This entity is a diagnosis of exclusion and requires ruling out primary causes such as exacerbation of congestive heart failure, aspiration pneumonia, pulmonary contusion, and other disease processes that may cause pulmonary edema formation. NPE has a characteristic, rapid formation of edema typically occurring in a few hours after the onset of CNS injury. Intracranial hypertension is common and the treatment should focus on promptly treating ICP and optimizing cerebral perfusion pressure while addressing the underlying brain injury. Since this is a multisystem failure involving brain, heart, lungs as well as the peripheral vasculature, care should be given to ensure euvolemic state, support the contractility and vascular tone, and resuscitate the brain all simultaneously. 

## 7. Pulmonary Embolism

**Figure 4:**
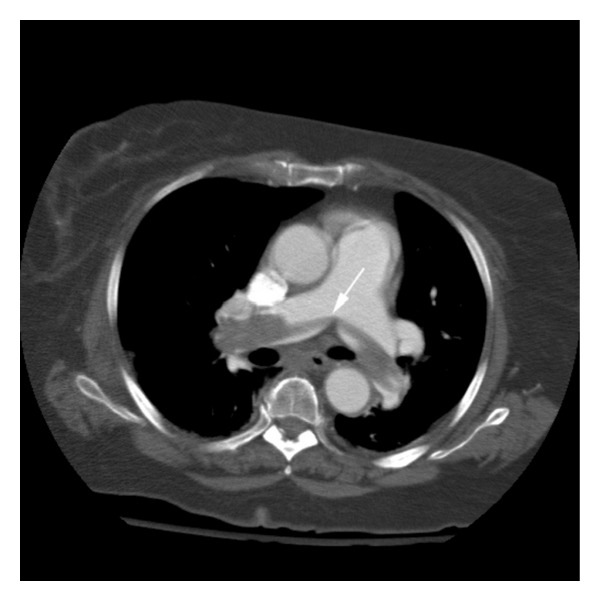
Saddle pulmonary embolism (white arrow) in a patient with SAH.

Venous thromboembolism (VTE) is a frequent and serious disease that encompasses both DVT and PE [54] (Figure 4). The epidemiology of deep venous thrombosis and pulmonary embolism in severe brain injured patients varies according to the population studied, injury severity, associated comorbidities and injuries, and the diagnostic methods. In trauma patients, the prevalence of DVT is 18–60% [55–57] and that of PE is 4–22% [58]. Studies in cohorts of ICH patients demonstrate a prevalence of 2% for PE and 1% for DVT [5]. In the SAH population, the prevalence of PE is < 1% and of DVT is 5–7% [2]. The effects of VTE may be detrimental for the critically-ill neurological patient, leading to postphlebitic syndrome, recurrent VTE, and potentially PE with a mortality rate of 9–50%. Clinical diagnosis of VTE is very difficult, and the sensitivity and specificity of clinical exam are very poor. Therefore, studying patients at higher risk or with higher prevalence of risk factors for the development of VTE requires use of invasive and noninvasive testing.

Even with the use of pneumatic compression devices, the higher incidence of DVT makes prophylactic heparin therapy desirable. Mechanical devices for DVT prophylaxis are considered to be a standard of care [59]. As opposed to pharmacological prophylaxis, mechanical devices may minimize hemorrhagic complications but may not sufficiently reduce the VTE rates. The adverse rates of hemorrhagic complications with pharmacological prophylaxis compared with the rates of VTE postprocedure are not well studied, and the optimal method of prophylaxis in neurosurgical patients (mechanical, pharmacological, or both) remains controversial. A recent study in neurosurgical patients showed that the majority of DVTs occurred within the first week after neurosurgical procedures and that the use of early subcutaneous heparin (at either 24 or 48 hours) was associated with a 43% reduction of developing a lower-extremity DVT, without an increase in surgical site hemorrhage without association of pharmacological prophylaxis with overall PE occurrence [59]. In general, severe brain injured patients benefit from early use of pharmacological prophylaxis for VTE. After craniotomy, low dose subcutaneous heparin (5000 U BID or TID) starting after the second day significantly reduces the frequency of venous thromboembolism, with no increase in intracranial bleeding [60]. Treatment with low molecular weight heparin (i.e., enoxaparin 40 mg daily) is a reasonable alternative if renal function is normal, and the results of recent studies suggest that both are equally effective with similar rates of heparin-induced thrombocytopenia [61]. When contraindicated, the use of inferior vena cava filters may be necessary in the short term [59].

## 8. Conclusions

Pulmonary complications are very prevalent in critically-ill neurological patients. Critical care specialists and professionals providing care for these patients must have a thorough understanding of their physiopathology, diagnostic methodologies, treatment alternatives, and overall impact on patient's outcomes.
